# Stress-induced anxiety-related behavior in mice is driven by enhanced excitability of ventral tegmental area GABA neurons

**DOI:** 10.3389/fnbeh.2024.1425607

**Published:** 2024-07-17

**Authors:** Eric H. Mitten, Anna Souders, Ezequiel Marron Fernandez de Velasco, Kevin Wickman

**Affiliations:** ^1^Graduate Program in Neuroscience, University of Minnesota, Minneapolis, MN, United States; ^2^Department of Pharmacology, University of Minnesota, Minneapolis, MN, United States

**Keywords:** anxiety, excitability, GABAA receptor, GABAB receptor, glutamate, shock, stress, VTA

## Abstract

**Introduction:**

Stress and trauma are significant risk factors for many neuropsychiatric disorders and diseases, including anxiety disorders. Stress-induced anxiety symptoms have been attributed to enhanced excitability in circuits controlling fear, anxiety, and aversion. A growing body of evidence has implicated GABAergic neurons of the ventral tegmental area (VTA) in aversion processing and affective behavior.

**Methods:**

We used an unpredictable footshock (uFS) model, together with electrophysiological and behavioral approaches, to investigate the role of VTA GABA neurons in anxiety-related behavior in mice.

**Results:**

One day after a single uFS session, C57BL/6J mice exhibited elevated anxiety-related behavior and VTA GABA neuron excitability. The enhanced excitability of VTA GABA neurons was correlated with increased glutamatergic input and a reduction in postsynaptic signaling mediated via GABA_A_ and GABA_B_ receptors. Chemogenetic activation of VTA GABA neurons was sufficient to increase anxiety-related behavior in stress-naïve mice. In addition, chemogenetic inhibition of VTA GABA neurons suppressed anxiety-related behavior in mice exposed to uFS.

**Discussion:**

These data show that VTA GABA neurons are an early substrate for stress-induced anxiety-related behavior in mice and suggest that approaches mitigating enhanced excitability of VTA GABA neurons may hold promise for the treatment of anxiety provoked by stress and trauma.

## Introduction

Stress and trauma are significant risk factors for a number of neuropsychiatric diseases, including anxiety disorders (Shin and Liberzon, 2010). The occurrence of post-traumatic stress disorder (PTSD) following a single traumatic event highlights the potent influence of stress in the etiology of clinically relevant anxiety (Yehuda et al., 2015). Acute stress exposure in mice is sufficient to induce anxiety symptomatology via several modalities, including restraint (Kemp et al., 2022; Liu et al., 2023; Chen et al., 2024) and inescapable shock (Tillage et al., 2020, 2021; McGovern et al., 2022). The effect of acute stress also translates to humans (Grillon et al., 2007; Daviu et al., 2019). Moreover, stress exposure in rodents can have a kindling effect, leading to more severe pathological symptoms over time (Rosen and Schulkin, 2022). Indeed, the number of experienced stressors correlates with subsequent presence of anxiety symptoms and incidence of diagnosis in humans (Gould et al., 2021; Schneider et al., 2021). Therefore, identifying neuronal substrates that are acutely sensitive to stress may shed light on how early anxiety symptoms can progress to more severe pathology.

Stress-induced anxiety symptoms have been attributed to enhanced excitability in circuits controlling fear, anxiety, and aversion (Rosen and Schulkin, 2022). Indeed, stress exposure in rodent models acutely engages and promotes excitability in the amygdala (Sarabdjitsingh et al., 2012; Zhang et al., 2021) and lateral habenula (LHb) (Brown and Shepard, 2013; Park et al., 2017). Ventral tegmental area (VTA) dopamine (DA) neurons, often associated with reward processing, have also been implicated in stress-induced pathological behavior in rodents (Holly and Miczek, 2016; Baik, 2020). Moreover, VTA DA neurons exhibit plasticity promoting increased activity following acute stress exposure (Niehaus et al., 2010; Graziane et al., 2013). However, the effect of stress on other VTA neuron subtypes has yet to be thoroughly investigated.

Emerging evidence suggests that VTA GABA neurons, key regulators of VTA DA neuron activity (Simmons et al., 2017; Polter et al., 2018), are involved in aversion processing and mediating the effects of stress. VTA GABA neurons exhibit reciprocal long-range projections to areas associated with fear, anxiety, and aversion that exhibit stress-induced hyperexcitability, including the amygdala and LHb (Taylor et al., 2014; Beier et al., 2015). VTA GABA neuron activity in mice increases in response to aversive or stressful stimuli, as well as the conditioned stimuli that predict them (Cohen et al., 2012; Root et al., 2020). Furthermore, VTA GABA neuron activity in mice can bi-directionally regulate real-time place preference and anxiety-related behavior (Tan et al., 2012; Jennings et al., 2013; Chen et al., 2020; Yu et al., 2021); activation of VTA GABA neurons is anxiogenic/aversive and inhibition is anxiolytic/appetitive.

In this study, we used an unpredictable footshock (uFS) protocol shown previously to increase anxiety-related behavior in C57BL/6J mice (Tillage et al., 2021) to investigate adaptations in VTA GABA neurons and to examine the relevance of VTA GABA neuron excitability to anxiety-related behavior. We found that a single unpredictable footshock (uFS) session in mice increases the excitability of VTA GABA neurons that is associated with adaptations in both excitatory and inhibitory neurotransmission. Furthermore, we demonstrate that chemogenetic activation of VTA GABA neurons is sufficient to promote anxiety-related behavior, and chemogenetic inhibition of VTA GABA neurons can suppress anxiety-related behavior provoked by uFS.

## Materials and methods

### Animals

All studies were approved by the Institutional Animal Care and Use Committee at the University of Minnesota. Male and female GAD67-GFP mice were provided by Takeshi Kaneko (Tamamaki et al., 2003) and GADCre(+) mice (B6J.Cg-Gad2^tm2(cre)Zjh^/MwarJ) were obtained from The Jackson Laboratory (#028867; Bar Harbor, ME). Both lines were maintained by perpetual backcrossing with C57BL/6J mice purchased from The Jackson Laboratory; at least 15 rounds of backcrossing had occurred before setting up breeding cages that produced the mice used in this study. Genotypes were determined using validated PCR-based genotyping protocols. All mice were group-housed throughout the study, with no more than 4 males or 5 females housed in a single cage. Mice were maintained on a 14:10 h light/dark cycle and were provided *ad libitum* access to food and water.

### Reagents

Kynurenic acid and baclofen were acquired from Sigma-Aldrich (Saint Louis, MO). Picrotoxin, CGP55845, and clozapine N-oxide (CNO) were acquired from Tocris (Bristol, UK). High titer (>1 × 10^12^ genocopies/mL) pAAV-hSyn-DIO-hM3Dq(mCherry) (RRID:Addgene_44361), pAAV-hSyn-DIO-hM4Di(mCherry) (RRID:Addgene_44362), and pAAV-hSyn-DIO-mCherry (RRID:Addgene_50459) plasmids, which were gifts from Bryan Roth (Krashes et al., 2011), were packaged in AAV8 by the University of Minnesota Viral Vector and Cloning Core (Minneapolis, MN) and stored at −80°C in single-use aliquots.

### Unpredictable footshock

We used an unpredictable footshock (uFS) protocol shown previously to induce anxiety-related behavior in male and female C57BL/6J mice (Tillage et al., 2021). uFS was delivered via an electric grid in sound-attenuated chambers containing a house light (product #: MED-VFC-USB-M, Med Associates, Inc.; Fairfax, VT) 1 d prior to electrophysiological or behavioral testing. Mice (7–9 wk) were given 20 footshocks (1 mA, 0.5 ms) randomly interspaced at 30, 60, or 90 s intervals during a single 20-min session. Boxes were cleaned with 70% ethanol prior to and between each session. Control animals were exposed to the same chamber for 20 min, but no footshocks were administered. All group-housed animals, including littermates not used for slice electrophysiological or behavioral experiments, experienced uFS or control conditions.

### Slice electrophysiology

Electrophysiological recordings were made in acutely isolated horizontal slices of the VTA from GAD67-GFP(+) mice, as described (DeBaker et al., 2023). All experiments were conducted in an oxygenated aCSF bath at 32–34°C. GFP-positive cells (GABA neurons) located medial to the medial terminal nucleus of the optic tract and lateral to midline nuclei in these slices (linear nuclei of the raphe or interpeduncular nuclei, when applicable) were targeted for analysis. Importantly, cells targeted for analysis were not located in the rostromedial tegmental nucleus. Drugs were applied via an 8-channel pinch valve perfusion system controlled by a ValveLink 8.3 controller (AutoMate Scientific, Inc.; Berkeley, CA). All measured and command potentials accounted for a −10 mV junction potential calculated using JPCalc software (Molecular Devices, LLC; San Jose, CA). Data were acquired using a MultiClamp 700A amplifier (Molecular Devices), low-pass filtered at 2 kHz, digitized at 10 kHz, and analyzed with pCLAMP v.9 software (Molecular Devices). Holding current and series resistance (R_s_) were measured before and after each recording. Experiments in which R_s_ exceeded 20 MΩ were removed.

Baseline excitability, spontaneous excitatory postsynaptic currents (sEPSCs), and baclofen-evoked currents were measured in an internal pipette solution consisting of (in mM): 140 K-methanesulfonate, 2 MgCl_2_, 1.1 EGTA, 5 HEPES, 2 Na_2_-ATP, 0.3 Na-GTP, and 5 phosphocreatine (pH = 7.2). Spontaneous firing rate was assessed over a 20-s interval immediately after whole-cell access was achieved. Rheobase was then acquired using 5 pA incremental steps starting at −60 pA. sEPSCs were measured at −70 mV in the presence of 100 μM picrotoxin to block GABA_A_R; kynurenic acid (2 mM) was used to assess the relevance of ionotropic glutamate receptors to observed events. Currents evoked by baclofen (200 μM) were measured at −60 mV in normal aCSF solution, and the GABA_B_R antagonist CGP55845 (2 μM) was used to test the relevance of GABA_B_R to observed responses. Spontaneous inhibitory postsynaptic currents (sIPSCs) were measured with an internal solution consisting of (in mM): 140 KCl, 2 MgCl_2_, 1.1 EGTA, 5 HEPES, 2 Na_2_-ATP, 0.3 Na-GTP, and 5 phosphocreatine (pH = 7.2). sIPSCs were recorded at a holding potential of −70 mV in the presence of 2 mM kynurenic acid to block ionotropic glutamate receptor currents and picrotoxin (100 μM) was used to assess the relevance of GABA_A_R to observed events.

### Intracranial viral manipulations

Procedures for intracranial viral infusion targeting the VTA were described previously (DeBaker et al., 2023). In brief, mice (>50 d) were placed in a stereotaxic frame (Model 1900, David Kopf Instruments; Tujunga, CA) under isoflurane anesthesia. Bilateral microinjectors consisting of a 33-gage stainless steel tube within a shorter 26-gage stainless steel tube were attached to polyethylene-20 tubing affixed to a 10 μL Hamilton syringe. Microinjectors were lowered through bilateral burr holes in the skull to the VTA (A/P: −2.75 mm, M/L: ±0.55 mm, D/V: −5.0 mm). AAV vectors (400 nL) were infused at a rate of 100 nL/min and microinjectors were left in place for 5 min to reduce backflow along the injection track upon removal. Behavioral experiments occurred 2–3 wk. after viral infusion.

### Behavioral testing

**Figure 1:**
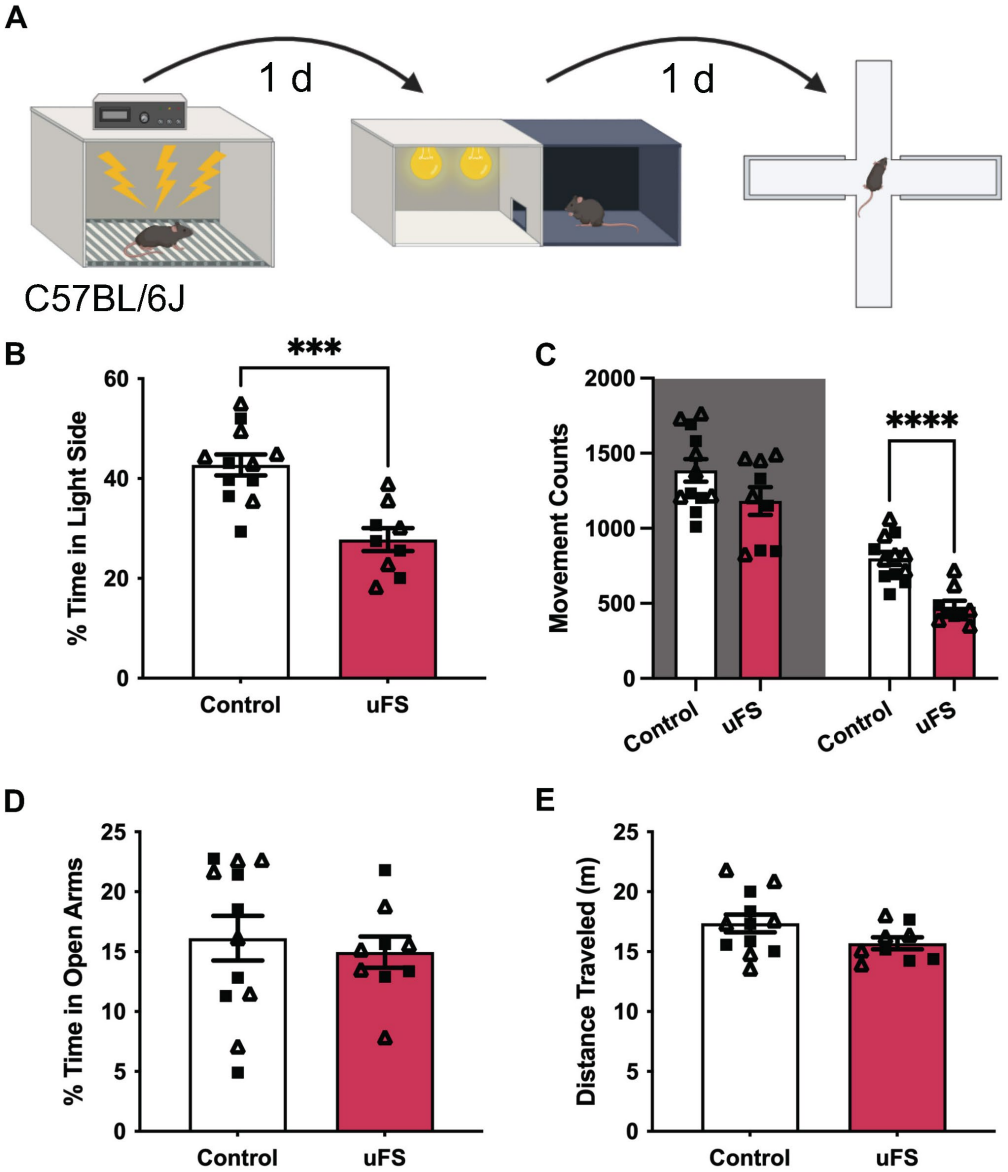
Unpredictable footshock (uFS) increases anxiety-related behavior in C57BL/6J mice. **(A)** Timeline of uFS and behavioral assessment in the light/dark box (LDB) test and elevated plus maze (EPM). **(B)** Percentage of time spent in the light side of the LDB in uFS-treated and control mice (*t*_19_ = 4.774, ***p = 0.0001; *n* = 9–12). **(C)** Movement counts in the dark (left, shaded; *t*_19_ = 1.723, *p* = 0.1011, *n* = 9–12) and light (right; *t*_19_ = 5.331, ****p < 0.0001, *n* = 9–12) side of the LBD in uFS-treated and control mice. **(D)** Percentage of time spent in the open arms of the EPM in uFS-treated and control mice (*t*_19_ = 0.4779, *p* = 0.6382, *n* = 9–12). **(E)** Distance traveled in the EPM in uFS-treated and control mice (*t*_19_ = 1.737, *p* = 0.0986, *n* = 9–12). All data are presented as mean ± SEM. Male and female datapoints are denoted by squares and triangles, respectively.

Each behavioral dataset was derived from a separate cohort of mice. All testing occurred during the light phase (0900–1,400 h). Subjects underwent 2 d of habituation prior to behavioral testing. On habituation days, mice were acclimated to the testing room for 1 h. During this time, mice were subjected to experimental handling (weighing, scruffing, moving around the room) for 2–3 min. Mice were exposed to uFS on Day 1, and either light/dark box (LDB) test or home cage locomotor activity was assessed on Day 2. For studies reported in Figure 1, elevated plus maze (EPM) was assessed on Day 3. On test days, mice were acclimated to the room for 1 h prior to handling/testing. The order of testing for individual subjects was randomized, and male and female mice were processed simultaneously.

The LDB test was conducted in a 2-compartment chamber (product #:ENV-3013-2, Med Associates, Inc.; Fairfax, VT) housed in sound-attenuating cubicles (product #: ENV-022-MD, Med Associates, Inc.; Fairfax, VT). Mice started the test in the dark compartment facing the open door to the light compartment. Time spent on each side of the compartment and movement counts were tracked for 15 min by infrared sensors. The percentage of time spent in the light compartment and total movement counts were extracted using MED-PC IV 4.2 software (Med Associates, Inc.; Fairfax, VT). For home cage locomotor behavior, mice were placed individually into new home cages containing used bedding from their original group-housed home cage and allowed to freely explore for 30 min. Activity was recorded via a video camera and distance traveled was extracted using ANY-maze 5.2 software (Stoelting Co.; Wood Dale, IL).

EPM was conducted on a raised, “plus-shaped” apparatus with two open arms and two closed arms (product#: ENV-560A, Med Associates, Inc.; Fairfax, VT). At the start of testing, animals were placed in the center of the maze, facing one of the open arms. Behavioral activity was recorded via a video camera for 5 min. The percentage of time spent in the open arms and total distance traveled were extracted utilizing ANY-maze 5.2 software (Stoelting Co.; Wood Dale, IL).

For chemogenetic manipulation studies, mice were given an IP injection of saline on habituation days and CNO (2 mg/Kg IP) was administered 30 min before each behavioral test. Testing involved 2 separate cohorts. The first cohort was exposed to EPM on Day 3. The second cohort was exposed to LDB testing on Day 3 and EPM on Day 5. No difference in EPM performance was detected between these 2 cohorts, so these data were combined. For the chemogenetic inhibition experiment, uFS occurred on Day 3 and LDB testing occurred on Day 4. The scope and accuracy of viral targeting was assessed in each subject after behavioral testing by analysis of viral-driven mCherry fluorescence in midbrain sections. Only mice that exhibited bilateral fluorescence confined primarily to the VTA were included in the final analysis.

### Statistical analyses

Data are presented throughout as mean ± SEM. All analyses were performed using Prism v.10 (GraphPad Software, Inc.; Boston, MA). Data points > 2 standard deviations from the mean were excluded from analysis; a total of 21 datapoints were excluded based on this approach. Male and female mice were used in all studies and groups were balanced by sex. The impact of sex on studied parameters was evaluated using 2-way ANOVA. No main effect of sex or an interaction was detected in any dataset. Therefore, data from male and female mice were pooled for analysis. Male and female datapoints are designated throughout by squares and triangles, respectively. Normality and lognormality tests were conducted to inform use of either the unpaired Student’s *t*-test or Mann–Whitney test. For all comparisons, differences were considered significant if *p* < 0.05.

## Results

### Unpredictable footshock increases anxiety-related behavior in C57BL/6J mice

We first assessed the efficacy of the unpredictable footshock protocol (uFS) in male and female C57BL/6J mice by assessing anxiety-related behavior in the light–dark box (LDB) and elevated plus maze (EPM) test, measured 1 d and 2 d after uFS, respectively (Figure 1A). No sex differences were observed related to the impact of uFS on behavioral performance and, as such, male and female data were combined. uFS-treated mice exhibited reduced time spent in the light side of the LDB box (Figure 1B), consistent with an increase in anxiety-related behavior. This effect correlated with a selective decrease in movement in the light side, as movement in the dark side did not differ across groups (Figure 1C). Similarly, uFS did not impact home cage locomotor activity, as assessed in a separate cohort of mice (Supplementary Figure S1). These findings suggest that uFS-induced decrease in time spent in the light side of the LDB is not due to a generalized decrease in activity. Interestingly, uFS did not impact time spent in the open arms (Figure 1D) or total distance traveled (Figure 1E) in the EPM. Thus, a single session of uFS enhances anxiety-related behavior in C57BL/6J mice in some (LDB) but not all (EPM) tests.

### Unpredictable footshock increases the excitability of VTA GABA neurons in mice

**Figure 2:**
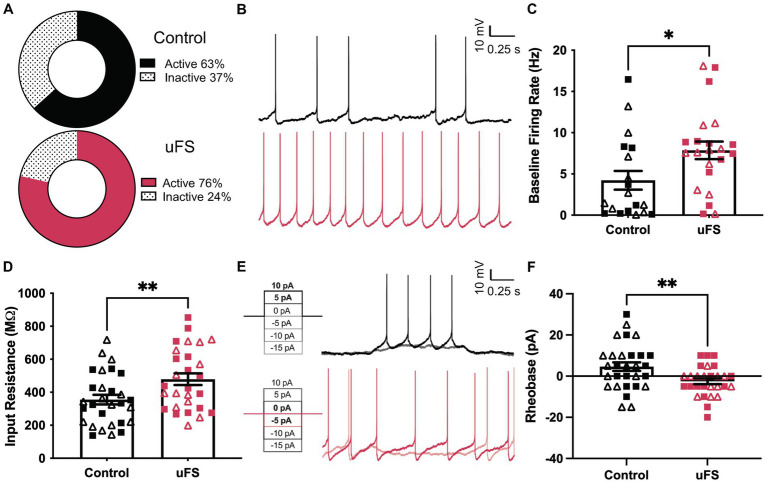
Enhanced excitability of VTA GABA neurons following unpredictable footshock (uFS). **(A)** Fraction of VTA GABA neurons showing spontaneous activity 1 d after uFS or control handling (*p* = 0.2549, *n* = 28–30). **(B)** Segments of traces from current-clamp experiments (*I* = 0) showing spontaneous activity of VTA GABA neurons 1 d after uFS or control treatment; scale: 10 mV/0.25 s. **(C)** Firing rate of VTA GABA neurons exhibiting spontaneous activity from uFS- and control-treated mice (*U* = 123, *p = 0.0238; *n* = 19–22). **(D)** Input resistance of VTA GABA neurons from uFS-treated and control mice (*t*_55_ = 2.708, **p = 0.0090; *n* = 28–30). **(E)** Rheobase traces of VTA GABA neurons from uFS (*I* = −5 and *I* = 0 shown) and control (*I* = 5 and *I* = 10 shown) treated mice; scale: 10 mV/0.25 s. **(F)** Rheobase of VTA GABA neurons in uFS- and control-treated mice (*t*_55_ = 2.855, **p = 0.0061; *n* = 28–30). All data are presented as mean ± SEM. Male and female datapoints are denoted by squares and triangles, respectively.

We used GAD67-GFP(+) mice to study the impact of uFS on VTA GABA neuron physiology (Tamamaki et al., 2003). One day after a single session of uFS, midbrain slices containing the VTA were prepared and the excitability of VTA GABA (GFP-positive) neurons was evaluated. No sex differences were observed related to the impact of uFS on any measure of VTA GABA neuron excitability. While the percentage of cells active at baseline was not significantly affected by uFS (Figure 2A), the firing rate of spontaneously active VTA GABA neurons from uFS-treated mice was higher than controls (Figures 2B,C). In addition, the input resistance of VTA GABA neurons from uFS-treated mice was higher than that of controls (Figure 2D), and rheobase was reduced by uFS (Figures 2E,F). Thus, a single session of uFS increases the excitability of VTA GABA neurons in mice.

### Unpredictable footshock promotes intrinsic and extrinsic adaptations in VTA GABA neurons

**Figure 3:**
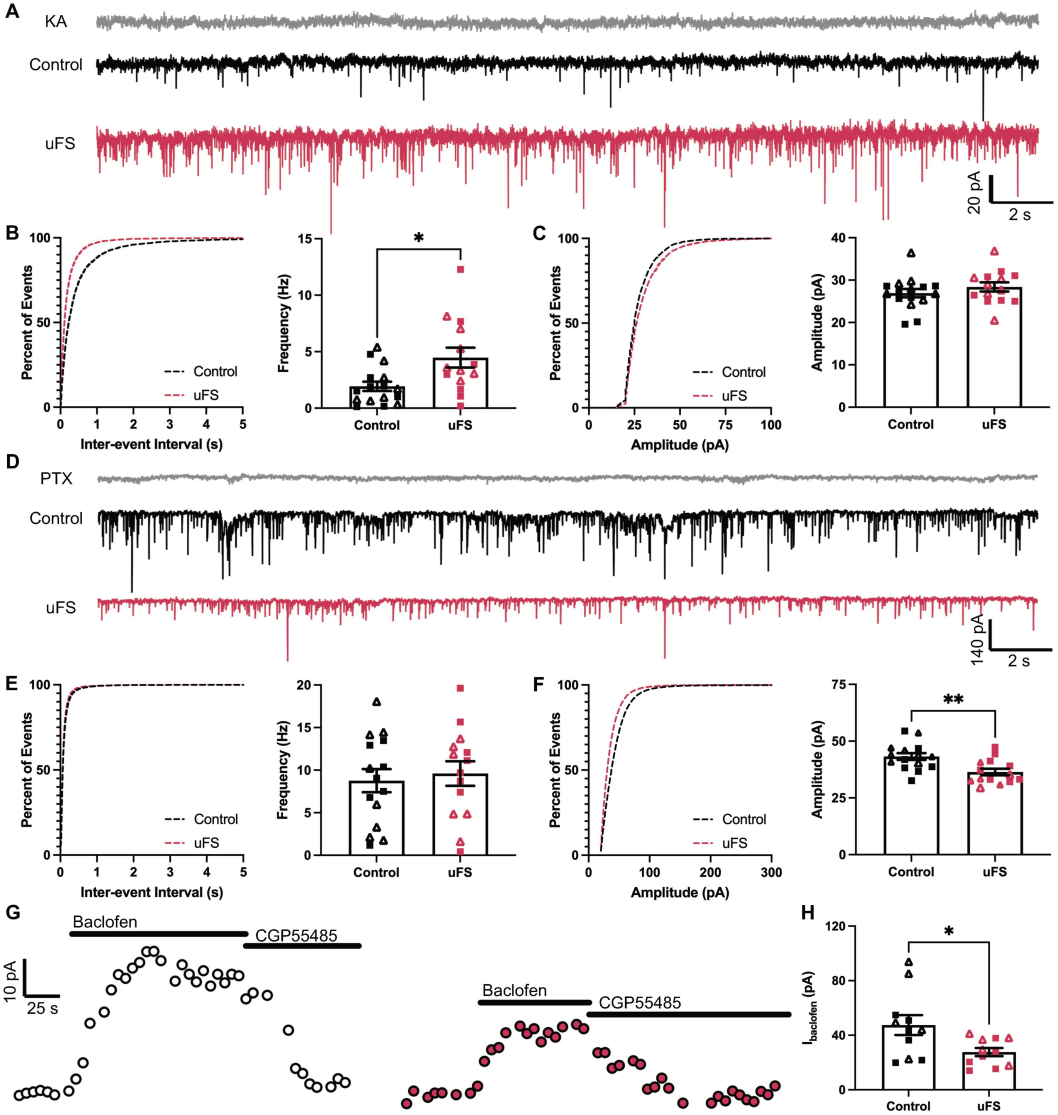
Adaptations evoked by unpredictable footshock (uFS) in VTA GABA neurons. **(A)** sEPSC traces measured 1 d after uFS or control handling; scale: 20 pA/2 s. Responses evoked under these conditions were reliably blocked by kynurenic acid (KA, 2 mM). **(B)** (Left) Cumulative distribution function for inter-event intervals of sEPSC events. (Right) sEPSC frequency in uFS-treated and control mice (*t*_28_ = 2.730, *p = 0.0108; *n* = 14–16). **(C)** (Left) Cumulative distribution function for amplitude of sEPSC events. (Right) sEPSC amplitude in uFS-treated and control mice (*t*_28_ = 1.017, *p* = 0.3180; *n* = 14–16). **(D)** sIPSC traces measured 1 d after uFS or control handling; scale: 140 pA/2 s. Responses evoked under these conditions were reliably blocked by picrotoxin (PTX, 100 μM). **(E)** (Left) Cumulative distribution function for inter-event intervals of sIPSC events. (Right) sIPSC frequency in uFS-treated and control mice (*t*_27_ = 0.4264, *p* = 0.6732; *n* = 14–15). **(F)** (Left) Cumulative distribution function for amplitudes of sIPSC events. (Right) sIPSC amplitude in uFS-treated and control mice (*t*_27_ = 3.234, **p = 0.0032; *n* = 14–15). **(G)** Outward currents (*V*_hold_ = −60 mV) evoked by baclofen (200 μM) measured 1 d following uFS or control handling; baclofen-evoked currents were reversed by CGP55845 (2 μM); scale: 10 pA/25 s. **(H)** Currents evoked by baclofen in uFS-treated and control mice (*t*_20_ = 2.519, *p = 0.0204; *n* = 11). All data are presented as mean ± SEM. Male and female datapoints are denoted by squares and triangles, respectively.

Stress-induced hyperexcitability in the LHb and amygdala has been associated with plasticity in signaling involving ionotropic glutamate (Li et al., 2011; Song et al., 2017; Authement et al., 2018) and GABA_A_ (Suvrathan et al., 2014; Langlois et al., 2022) receptors, ligand-gated ion channels that mediate fast excitatory and inhibitory neurotransmission, respectively. Therefore, we measured spontaneous excitatory (sEPSC) and inhibitory (sIPSC) postsynaptic currents in VTA GABA neurons 1 d after uFS or control handling. No sex differences were observed related to the impact of uFS on sEPSCs or sIPSCs. uFS increased the frequency (Figures 3A,B) but not amplitude (Figures 3A,C) of kynurenic acid-sensitive sEPSCs, consistent with increased glutamatergic input to VTA GABA neurons following uFS exposure. While uFS did not alter the frequency of sIPSCs in VTA GABA neurons (Figures 3D,E), it did reduce sIPSC amplitude (Figures 3D,F). As sIPSCs were blocked completely by picrotoxin, these findings suggest that uFS exposure in mice decreases the expression and/or activity of GABA_A_ receptors (GABA_A_R) in VTA GABA neurons.

Aversive experience including footshock has been shown to suppress signaling mediated by somatodendritic/postsynaptic GABA_B_ receptors (GABA_B_Rs) in LHb neurons (Lecca et al., 2016). GABA_B_Rs are inhibitory G protein-coupled receptors that regulate the activity of multiple enzymes and ion channels, contributing to the inhibitory influence of GABA on neuronal excitability (Rose and Wickman, 2022). Thus, we also tested whether uFS impacted the GABA_B_R-dependent somatodendritic inhibitory current in VTA GABA neurons. Whole-cell/somatodendritic inhibitory currents evoked by the GABA_B_R agonist baclofen were smaller in VTA GABA neurons from uFS-treated mice (Figures 3G,H). Collectively, these data suggest that intrinsic (increased input resistance, as well as diminished GABA_A_R and GABA_B_R responses) and extrinsic (increased glutamatergic input) adaptations provoked by uFS contribute to the enhanced excitability of VTA GABA neurons.

### VTA GABA neuron excitability bi-directionally impacts anxiety-related behavior

**Figure 4:**
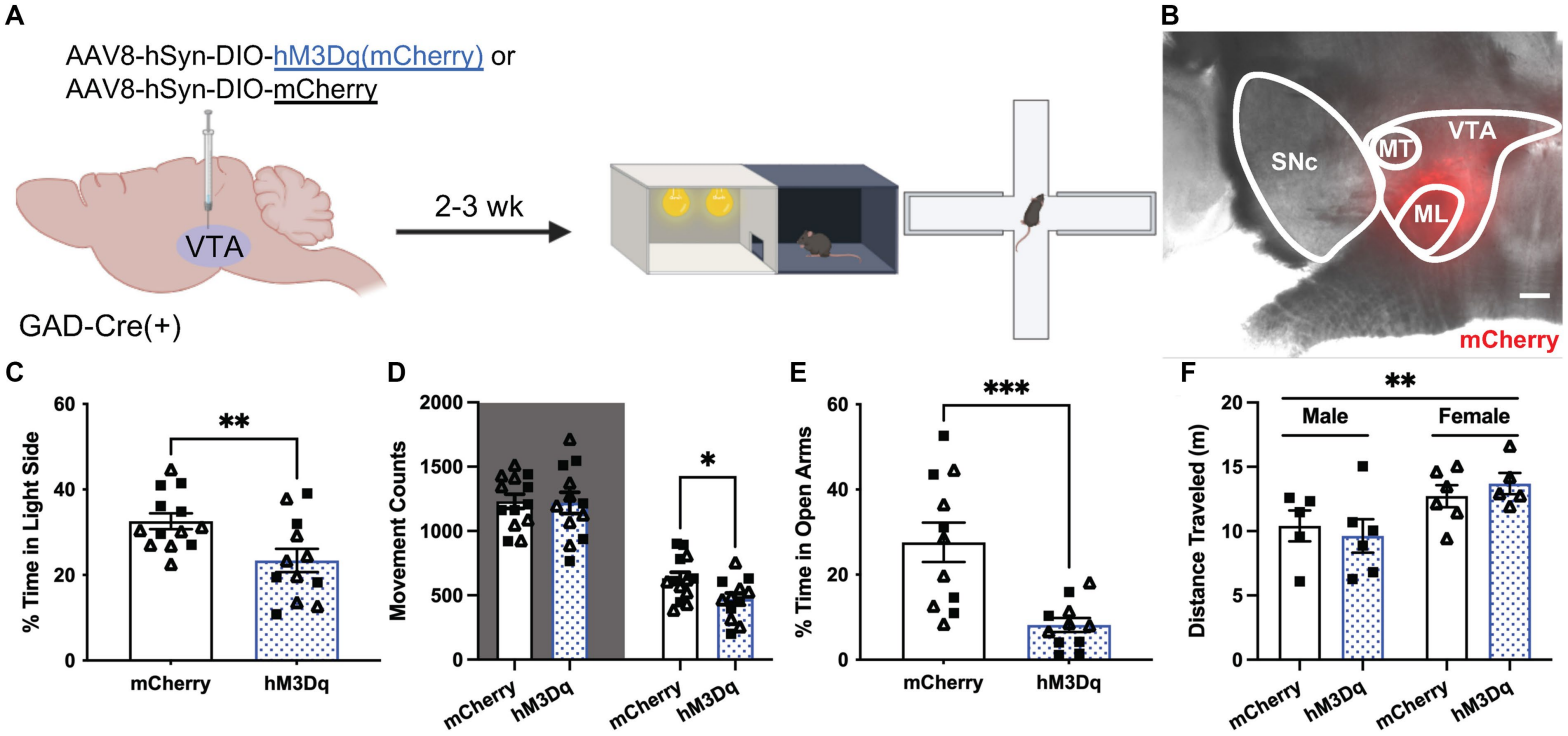
Chemogenetic excitation of VTA GABA neurons induces anxiety-related behavior. **(A)** Viral treatment and behavioral timeline for chemogenetic activation of VTA GABA neurons during the light/dark box (LDB) test or elevated plus maze in uFS-naïve mice. **(B)** Image of mCherry fluorescence associated with AAV8-hSyn-DIO-hM3Dq(mCherry) infusion in a horizontal slice of the VTA of a GADCre(+) mouse; scale = 200 μm. **(C)** Percentage of time spent in the light side of the LDB test 30 min after injection of clozapine N-oxide (CNO; 2 mg/Kg IP) from mCherry- and hM3Dq-expressing mice (*t*_23_ = 2.819, **p = 0.0097; *n* = 12–13). **(D)** Number of movement counts in the dark side (left, shaded; *t*_23_ = 0.1298, *p* = 0.8979; *n* = 12–13) and the light side (right; *t*_23_ = 2.437, *p = 0.0229, *n* = 12–13) for mCherry- and hM3Dq-expressing mice. **(E)** Percentage of time spent in the open arms of the EPM 30 min after injection of CNO (2 mg/Kg IP) from mCherry- and hM3Dq-expressing mice (*t*_20_ = 3.942, ***p = 0.0008; *n* = 11). **(F)** Total distance traveled in the EPM from mCherry and hM3Dq-expressing mice; two-way ANOVA revealed a main effect of sex (*F*_1, 18_ = 8.690, **p = 0.0086), with no main effect of treatment (*F*_1, 18_ = 0.0081, *p* = 0.9290) or interaction (*F*_1, 18_ = 0.6673, *p* = 0.4247) detected. All data are presented as mean ± SEM. Male and female datapoints are denoted by squares and triangles, respectively.

To examine the effect of VTA GABA neuron excitability on anxiety-related behavior, we used a chemogenetic approach to permit the acute excitation of VTA GABA neurons. GADCre(+) mice were treated with intra-VTA AAV8-hSyn-DIO-hM3Dq(mCherry) or control vector (AAV8-hSyn-DIO-mCherry) and tested 2–3 wk. later in LDB and EPM tests (Figures 4A,B). Treatment with CNO before testing reduced the percentage of time spent in the light side of the LDB test in hM3Dq-expressing mice relative to control subjects (Figure 4C). This was associated with a selective reduction in movement within the light compartment (Figure 4D). CNO treatment also reduced the time spent in the open arms of the EPM in hM3Dq-expressing mice relative to controls (Figure 4E). A main effect of sex was detected in total distance traveled in the EPM, but there was no effect of viral treatment or interaction between sex and viral treatment (Figure 4F). No sex differences were observed in any other behavioral measure. Thus, acute VTA GABA neuron activation is sufficient to increase anxiety-related behavior in LDB and EPM tests in stress-naïve mice.

**Figure 5:**
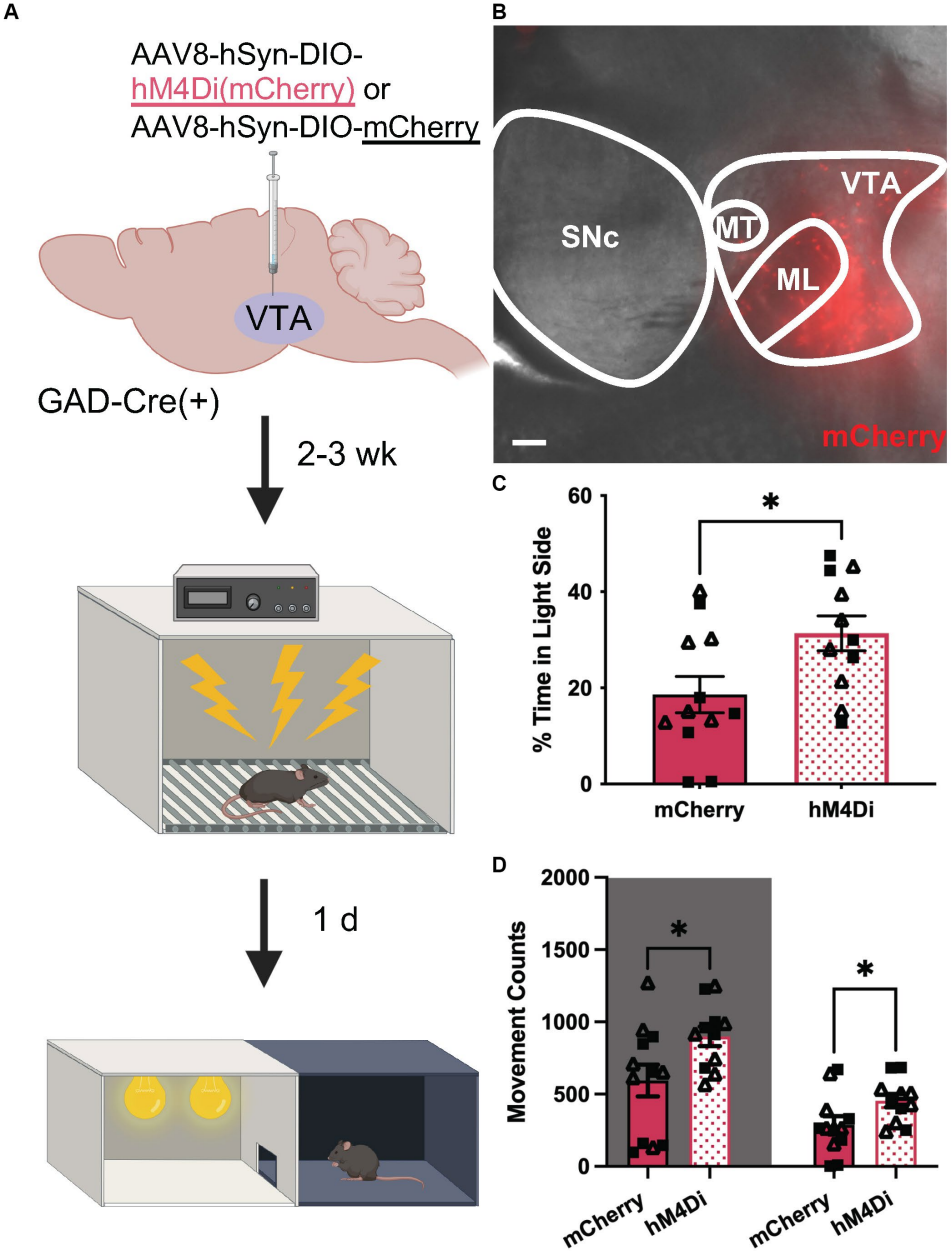
Chemogenetic inhibition of VTA GABA neurons attenuates uFS-induced anxiety-related behavior. **(A)** Viral treatment and behavioral timeline for chemogenetic inhibition of VTA GABA neurons during the LDB test 1 d following uFS treatment. **(B)** Image of mCherry fluorescence associated with AAV8-hSyn-DIO-hM4Di(mCherry) infusion in a horizontal slice of the VTA of a GADCre(+) mouse; scale = 200 μm. **(C)** Percentage of time spent in the light side 30 min after injection of CNO (2 mg/kg IP) for mCherry- and hM4Di-expressing mice (*t*_21_ = 2.421, *p = 0.0246; *n* = 11–12). **(D)** Number of movement counts in the dark side (left, shaded; *t*_21_ = 2.305, *p = 0.0315; *n* = 11–12) and the light side (right; *t*_21_ = 2.194, *p = 0.0396, *n* = 11–12) for mCherry- and hM4Di-expressing mice. All data are presented as mean ± SEM. Male and female datapoints are denoted by squares and triangles, respectively.

Finally, we tested whether chemogenetic inhibition of VTA GABA neurons could suppress anxiety-related behavior in uFS-treated mice. GADCre(+) mice were treated with intra-VTA AAV8-hSyn-DIO-hM4Di(mCherry) or control vector (AAV8-hSyn-DIO-mCherry) and were exposed to uFS after a 2–3 wk. recovery period (Figures 5A,B). As uFS increased anxiety-related behavior in the LDB but not EPM test (Figure 1), the effect of chemogenetic inhibition of VTA GABA neurons on uFS-induced anxiety-related behavior was only evaluated in the LDB test. Treatment with CNO before testing increased the time spent in the light side of the LDB test in hM4Di-expressing mice relative to controls (Figure 5C). This effect was associated with increased movement in both the dark and light sides of the LDB (Figure 5D). These data suggest that enhanced excitability of VTA GABA neurons is necessary for the expression of uFS-induced anxiety-related behavior in mice.

## Discussion

In this study, we investigated the impact of unpredictable footshock (uFS) on the excitability of VTA GABA neurons, as well as anxiety-related behaviors, in mice. We detected multiple adaptations that likely contribute to the enhanced excitability of VTA GABA neurons, including increased input resistance, increased glutamatergic input, and decreased postsynaptic GABA_A_R and GABA_B_R-dependent responses. Moreover, we found that chemogenetic activation of VTA GABA neurons is sufficient to induce anxiety-related behavior in stress-naïve mice, whereas chemogenetic inhibition of VTA GABA neurons can suppress the uFS-induced increase in anxiety-related behavior. Thus, enhanced VTA GABA neuron excitability is necessary for the expression of uFS-induced anxiety-related behavior.

### Increased presynaptic glutamatergic input to VTA GABA neurons

uFS exposure increased sEPSC frequency in VTA GABA neurons, consistent with an increase in glutamatergic input. While the source(s) of increased glutamatergic input is unclear, the LHb is an intriguing candidate as it provides a prominent glutamatergic input to VTA GABA neurons (Omelchenko et al., 2009). The LHb has been implicated in depression-related behavior (Browne et al., 2018), but preclinical and clinical evidence suggest that it also plays a role in stress-induced anxiety-related behavior (Jacinto et al., 2017; Ma et al., 2020; Tong et al., 2024). Glutamatergic neurons of the VTA are another potential candidate as these neurons are also activated by aversive stimuli and have been implicated in inescapable tailshock-induced anxiety-related behavior, as assessed by the LDB test (McGovern et al., 2022).

### Diminished postsynaptic GABA_A_R and GABA_B_R-dependent signaling in VTA GABA neurons

Preclinical and clinical studies have linked GABA_A_R-dependent signaling to anxiety symptomatology. Activation of GABA_A_R is anxiolytic, and drugs targeting GABA_A_R are used as treatments for anxiety disorders (Chen et al., 2019). Restraint, social defeat, and conditioned shock stress can decrease GABA_A_R-dependent current amplitude in the amygdala (Suvrathan et al., 2014; Botta et al., 2015; Qin et al., 2022). Here, we show that after uFS, VTA GABA neurons exhibit reduced sIPSC amplitude, indicative of decreased postsynaptic GABA_A_R activity. Interestingly, Gq signaling via G protein-coupled serotonin receptors can decrease GABA_A_R-dependent current amplitude in neurons (Feng et al., 2001; Wang et al., 2016). Moreover, stress exposure acutely engages dorsal raphe nucleus serotonergic neurons (Hale et al., 2012), which make reciprocal projections to VTA GABA neurons (Beier et al., 2015; Rahaman et al., 2022), highlighting a potential role for serotonergic signaling in the uFS-induced suppression of GABA_A_R activity.

Somatodendritic GABA_B_R-dependent inhibitory currents in VTA GABA neurons were also diminished by uFS. GABA_B_R is also connected to anxiety symptomatology; GABA_B_R agonists and positive allosteric modulators evoke anxiolysis in preclinical and clinical settings (Felice et al., 2022) and knockout of GABA_B_R subunits correlates with increased anxiety-related behavior in mice (Mombereau et al., 2004, 2005). Interestingly, footshock stress diminished GABA_B_R-induced currents in mouse LHb neurons (Lecca et al., 2016, 2017), and *in vivo* methamphetamine exposure in mice suppressed GABA_B_R-induced currents in VTA GABA neurons (Padgett et al., 2012). Both neuroadaptations were linked to GABA_B_R internalization and were rescued by inhibition of protein phosphatase 2A (PP2A) activity (Padgett et al., 2012; Lecca et al., 2016, 2017). Thus, uFS may provoke a similar PP2A-dependent, GABA_B_R internalization in VTA GABA neurons.

### Stress-induced affective behaviors in mice and VTA GABA neurons

uFS exposure induced anxiety-related behavior in the LDB but not EPM test. Shock stress-induced enhancement of anxiety-related behavior in the EPM has been demonstrated using protocols that include a habituation period to the shock context prior to shock administration and re-exposure to the shock context (Korte and De Boer, 2003; Girardi et al., 2013). Protocols that include these components have been proposed to model aspects of post-traumatic stress disorder by strengthening the association of the context to the shock exposure (Bali and Jaggi, 2015). A more intense shock stress protocol that included these aforementioned behavioral components was sufficient to induce anxiety-related behavior in both LDB and EPM tests (Zhang et al., 2018). Therefore, the uFS protocol used in this study may constitute a mild stressor, evoking a selective anxiogenic effect in the LDB test without affecting EPM performance.

VTA GABA neuron activity in mice tracks aversive stimuli and the conditioned stimuli that predict them (Cohen et al., 2012; Root et al., 2020; Lowes et al., 2021). Here, we show that chemogenetic activation of VTA GABA neurons enhances anxiety-related behavior in mice, as assessed by LDB and EPM tests. This aligns with previous optogenetic and chemogenetic experiments in stress-naïve mice demonstrating that VTA GABA neuron activity can modulate anxiety-related behavior (Chen et al., 2020; Yu et al., 2021) and can induce fear responses (Zhou et al., 2019) and real-time place aversion (Tan et al., 2012; Li et al., 2019). Collectively, these insights add to a growing body of evidence that VTA GABA neurons are a crucial hub of aversion processing and stress-induced affective behavior.

hM4Di inhibition of VTA GABA neurons decreased anxiety-related behavior in uFS-treated mice, as evidenced by an increase in the percentage of time spent in the light side of the LDB test. This inhibition also produced an increase in movement counts in both the dark and light sides of the box, which was expected due to previous evidence showing that hM4Di inhibition of VTA GABA neurons increases motor activity in mice exploring an open field (Yu et al., 2021). Additionally, a large body of evidence supports a link between VTA DA neuron activity and movement (Lerner et al., 2021), suggesting that VTA GABA neuron inhibition of VTA DA neurons could be provoking this non-selective increase in movement in hM4Di-expressing mice.

Activation and enhanced excitability of VTA GABA neurons following uFS could affect stress-induced anxiety-related behavior via long-range GABAergic projections to structures associated with fear, anxiety, and aversion (Taylor et al., 2014; Beier et al., 2015). The influence of VTA GABA neuron excitability may also be indirect, via inhibition of DA neurons (Bouarab et al., 2019). Indeed, inhibition of VTA DA neurons that project to the amygdala drives nicotine-induced anxiety-related behavior in mice (Nguyen et al., 2021), as well as social defeat stress-induced anxiety-related behavior (Morel et al., 2022). Acute stress exposure also promotes long-term potentiation in VTA DA neurons (Niehaus et al., 2010; Graziane et al., 2013), and it induces a transient increase in DA release in downstream projections of VTA DA neurons (Cabib and Puglisi-Allegra, 2012). Interestingly, *in silica* experiments have posited that synchronous activation of VTA GABA neurons can promote sustained firing of VTA DA neurons in the presence of elevated glutamate (Morozova et al., 2016). Moreover, genetic ablation of NMDAR in DA neurons, which selectively blunts phasic activity of VTA DA neurons, impairs aversive conditioning and promotes a generalized anxiety-related phenotype (Zweifel et al., 2011), suggesting that disruption of phasic VTA DA firing can contribute to anxiety-related behavior.

### Role of VTA GABA neuron subtypes

Chemogenetic excitation of VTA GABA neurons increased anxiety-related behavior in LDB and EPM tests, whereas uFS only increased anxiety-related behavior in the LDB test. This divergence may indicate that chemogenetic excitation of VTA GABA neurons evokes a stronger enhancement of VTA GABA neuron excitability than uFS. Alternatively, uFS may provoke adaptions in a subpopulation of VTA GABA neurons. In support of this contention, we found that uFS did not increase the percentage of VTA GABA neurons exhibiting spontaneous activity, but it did increase the rate of VTA GABA neurons that did exhibit spontaneous activity. The VTA is host to GABA neuron subtypes that can be differentiated based on molecular and neurochemical markers, as well as afferent and efferent connections (Paul et al., 2018; Bouarab et al., 2019; Kim and Kaang, 2022). Our electrophysiological assessments and chemogenetic manipulations of VTA GABA neurons did not discriminate among VTA GABA neuron subtypes. It is tempting to speculate that spontaneous activity of VTA GABA neurons could be a physiological biomarker of the subpopulation of GABA neurons that mediate stress response and associated anxiety-related behavior.

In conclusion, we show that VTA GABA neurons in mice exhibit enhanced excitability following a single session of uFS. This neuroadaptation is likely attributable to multiple intrinsic and extrinsic mechanisms, including enhanced glutamatergic input and concurrent suppression of postsynaptic GABA_A_R- and GABA_B_R-dependent signaling. Enhanced excitability of VTA GABA neurons was necessary for expression of uFS-induced anxiety-related behavior. Collectively, our data suggest that VTA GABA neurons are acutely sensitive to stress exposure and may be an important early target in the etiology of stress-related anxiety disorders. Developing therapeutic approaches that mitigate VTA GABA neuron excitability may hold promise for treatment of anxiety symptomatology induced by aversive and traumatic experience.

## Data availability statement

The raw data supporting the conclusions of this article will be made available by the authors, without undue reservation.

## Ethics statement

The animal study was approved by University of Minnesota Institutional Animal Care and Use Committee. The study was conducted in accordance with the local legislation and institutional requirements.

## Author contributions

ErM: Conceptualization, Data curation, Formal analysis, Investigation, Methodology, Validation, Writing – original draft, Writing – review & editing. AS: Investigation, Methodology, Validation, Writing – review & editing. EzM: Methodology, Resources, Validation, Writing – review & editing. KW: Conceptualization, Funding acquisition, Project administration, Resources, Supervision, Writing – original draft, Writing – review & editing.
