# Supplementary material for: Stress-induced anxiety-related behavior in mice is driven by enhanced excitability of ventral tegmental area GABA neurons
Source: Front Behav Neurosci. 2024 Jul 17;18:1425607. doi: 10.3389/fnbeh.2024.1425607 (PMC11288924; doi:10.3389/fnbeh.2024.1425607)
Supplement: Supplementary file 1 [file Data_Sheet_1.docx]

**Supplementary Figure 1. Home cage locomotor activity is unaffected by unpredictable footshock stress (uFS)**

**A**) Timeline of uFS and behavioral assessment in the home cage of a dedicated cohort of C57BL/6J mice.

**B**) Distance travelled in the home cage context 1 d following uFS in C57BL/6J mice (t_10_=1.864, p=0.0919, n=6). Male and female datapoints are delineated by squares and triangles, respectively.

**C**) Average speed in the home cage context 1 d following uFS in C57BL/6J mice (t_10_=1.869, p=0.0912, n=6)

All data are presented as mean ± SEM. Male and female datapoints are denoted by squares and triangles, respectively.
